# Mutation of *rpoB* Shifts the Nutrient Threshold Triggering *Myxococcus* Multicellular Development

**DOI:** 10.3389/fmicb.2022.817080

**Published:** 2022-03-03

**Authors:** Sabrina A. Eisner, Gregory J. Velicer, Yuen-Tsu N. Yu

**Affiliations:** Department of Environmental Systems Science, Institute of Integrative Biology, ETH Zurich, Zurich, Switzerland

**Keywords:** pleiotropy, stringent response, development, nutrient sensing, *rpoB*

## Abstract

The ability to perceive and respond to environmental change is essential to all organisms. In response to nutrient depletion, cells of the soil-dwelling δ-proteobacterium *Myxococcus xanthus* undergo collective morphogenesis into multicellular fruiting bodies and transform into stress-resistant spores. This process is strictly regulated by gene networks that incorporate both inter- and intracellular signals. While commonly studied *M. xanthus* reference strains and some natural isolates undergo development only in nutrient-poor conditions, some lab mutants and other natural isolates commit to development at much higher nutrient levels, but mechanisms enabling such rich medium development remain elusive. Here we investigate the genetic basis of rich medium development in one mutant and find that a single amino acid change (S534L) in RpoB, the β-subunit of RNA polymerase, is responsible for the phenotype. Ectopic expression of the mutant *rpoB* allele was sufficient to induce nutrient-rich development. These results suggest that the universal bacterial transcription machinery bearing the altered β-subunit can relax regulation of developmental genes that are normally strictly controlled by the bacterial stringent response. Moreover, the mutation also pleiotropically mediates a tradeoff in fitness during vegetative growth between high vs. low nutrient conditions and generates resistance to exploitation by a developmental cheater. Our findings reveal a previously unknown connection between the universal transcription machinery and one of the most behaviorally complex responses to environmental stress found among bacteria.

## Introduction

Sensing and responding to varying nutrient availability are crucial to all forms of life. Cells have thus evolved diverse molecular pathways to transduce nutrient signals from the environment and regulate gene expression to optimize responses to environmental change. In eukaryotes, for example, widely conserved TOR kinases are the most important regulators of cellular responses to nutrient changes (Cardenas et al., 1999). Among prokaryotes, a broadly conserved mechanism for responding to nutrient limitation is the stringent response, which was first discovered in *Escherichia coli* and has since been found throughout the bacterial domain (Chatterji and Kumar Ojha, 2001; Boutte and Crosson, 2013; Shimizu, 2014). In the stringent response, guanosine-5′-(tri)di-3′-diphosphate ((p)ppGpp) accumulates in response to amino acid starvation and causes major transcriptional changes that promote survival under stress. These changes include inhibition of stable rRNA synthesis and increased transcription of amino acid biosynthetic operons and stress-adaptive genes (Chatterji and Kumar Ojha, 2001; Diodati et al., 2014; Sharma et al., 2021).

Despite being highly conserved in its core elements, the stringent response has been evolutionarily integrated with a highly diverse array of downstream pathways (Boutte and Crosson, 2013; Irving and Corrigan, 2018). One of the most sophisticated bacterial behaviors controlled by the stringent response is found among the Gram-negative myxobacteria, in which accumulation of (p)ppGpp is required to initiate multicellular development upon amino acid deprivation, leading to fruiting body formation and spore differentiation (Manoil and Kaiser, 1980; Harris et al., 1998). Studies of *Myxococcus xanthus* have shown that myxobacterial fruiting body development is orchestrated by several intercellular signals and chemosensory pathways ultimately triggered by the stringent response (Zusman et al., 2007).

*Myxococcus xanthus* is a socially motile and predatory bacterium (Hartzell and Youderian, 1995; Keane and Berleman, 2016) that grows vegetatively when prey or other macromolecular nutrients are abundant. As nutrient levels decrease, *M. xanthus* faces the decision whether to continue vegetative growth or initiate its developmental program (Kaiser, 2004). When nutrient levels drop below a threshold, the stringent response activates early stages of development-specific gene expression (Singer and Kaiser, 1995; Kaplan and Plamann, 1996; Crawford and Shimkets, 2000). *Myxococcus xanthus* natural isolates have diversified greatly with respect to the nutrient threshold that triggers development, but the genetic and evolutionary causes of this diversity remain unknown (Kraemer et al., 2010).

Many factors are required for *M. xanthus* to initiate development, including a solid surface allowing either or both of two motility systems to drive aggregation (Kroos et al., 1988; Hartzell and Youderian, 1995), a minimum cell density (Shimkets and Dworkin, 1981; Kaplan and Plamann, 1996) and perception of a nutrient downshift (Dworkin, 1963; Diodati et al., 2014). Cell-density detection is mediated by an extracellular quorum-sensing signal known as A-signal, which involves several known genes (e.g., *asgA*, *asgB*, and *asgC*) necessary for early mound formation (Kuspa and Kaiser, 1989). In A-signaling, the response to starvation involves protease degradation of surface proteins to amino acids and short peptides that collectively constitute the A-signal, which triggers development at sufficiently high concentrations (Guo et al., 2000). However, production of A-signal is itself regulated by the stringent response (Harris et al., 1998), as is expression of other genes regulating early steps of the developmental process. Additional genes known to be involved in *M. xanthus* nutrient sensing include *socE*, *nsd*, the *che3* operon, *sigC*, and *Mxan_2902* (a σ54-type transcriptional activator; Apelian and Inouye, 1993; Crawford and Shimkets, 2000; Diodati et al., 2014).

Another important regulator controlling the *M. xanthus* response to nutrient deprivation is a non-coding small RNA (sRNA) unique to the myxobacteria—Pxr—that prevents development from initiating when nutrients are abundant (Yu et al., 2010; Chen et al., 2014). In essence, Pxr controls a checkpoint that modulates the transition from vegetative growth to development. Inactivation of Pxr function by either mutation or deletion bypasses the checkpoint and thereby allows spore formation to occur on nutrient-rich medium (Yu et al., 2016, 2017). Mutations in other genes known to be part of the Pxr regulatory pathway show the same phenotype of rich medium (RM) development (Yu et al., 2016).

In a preliminary experiment performed while investigating the mechanistic basis of RM development, we sought to test whether a *pxr* deletion mutant (GJV1*pxr*) proficient at RM development produces extracellular molecules that can socially induce the wild-type parent strain GJV1 to also undergo development in a mixed population of both strains on nutrient-rich agar medium. An unexpectedly high proportion of spores formed on rich medium in this experiment were formed by GJV1, suggesting that RM development by GJV1 can be externally stimulated by the *pxr* deletion mutant. Five isolates of GJV1-derived colonies grown from spores in this experiment were examined for the RM development phenotype in pure culture to test whether their founding spores in the prior experiment were formed due to extracellular complementation by GJV1*pxr* or might rather have been formed due to a spontaneous mutation in the GJV1 background that confers the RM development phenotype. Only one of the clones—designated YTY2 (Supplementary Table S1)—showed a strong RM development phenotype even in the absence of GJV1*pxr*, indicating that a mutation in YTY2 is responsible for its RM development. Here we identify the mutation responsible for the YTY2 RM development phenotype, test for several potential pleiotropic phenotypes and consider the implications of our results for the roles of *rpoB* in the general bacterial stringent response and in regulating the onset of fruiting body development in the myxobacteria.

## Materials and Methods

### Strains, Media, and Reagents

Strains described in this study are listed in Supplementary Table S1. Luria-Bertani (LB) liquid medium or LB plates containing 1.5% Bacto agar were used to cultivate *E. coli* and its derivatives. For vegetative growth, *M. xanthus* was cultivated in CTT liquid [8 mM MgSO_4_, 10 mM Tris–HCl (pH 8.0), 1% Bacto casitone, 1 mM KH_2_PO_4_] or on CTT plates containing 1.5% Bacto agar [hard agar (HA)] or 0.5% Bacto agar [soft agar (SA)]. For starvation and RM development, *M. xanthus* was incubated on TPM agar [8 mM MgSO_4_, 10 mM Tris–HCl (pH 8.0), 1 mM KH_2_PO_4_ 1.5% Bacto agar] and 0.3%-casitone CTT-HA (containing 3 g/L Bacto casitone, which is 30% of the casitone level in standard CTT medium), respectively. Kanamycin (40 μg/ml) and oxytetracycline (12.5 μg/ml) were used where stated. Galactose (1.5%) was used for selection for allelic exchange. Bacterial growth was assessed by measuring OD_595_ with a TECAN plate reader (Infinite M200 PRO).

### Plasmid Construction

Mutations were identified by sequencing strain YTY2 on an Illumina® platform and alignment of the resulting reads to the parental genome GJV1 (Rendueles et al., 2015; Nair et al., 2019). The DNA fragments of interest were amplified from GJV1 genomic DNA by PCR using the Phusion DNA polymerase and the primers listed in Supplementary Table S2. Each set of primers was designed to create a PCR product that contained equivalently sized flanking regions on each side of the mutation of interest. PCR products of expected sizes were purified from a 1% agarose gel using a QIAquick Gel Extraction Kit (QIAGEN) and ligated into a pCR-Blunt vector to generate pCR-0795, pCR-*rpoB*, and pCR-6547. Each construct was digested with EcoRI to confirm the insert of the expected size and was sequenced to verify the intended genetic elements. Subsequently, the inserts from pCR-0795, pCR-*rpoB*, and pCR-6547 were isolated following digestion with BamHI-HF and EcoRV-HF. The inserts were then ligated into pBJ113-*cglB* that had been linearized with BamHI and HincII, resulting in pBJ-0795, pBJ-*rpoB*, and pBJ-6547 (Supplementary Table S3). All pBJ113 derivatives contained a kanamycin-resistance cassette and a galactokinase (*galK*) gene that was strategically important for allelic exchange. To construct the pVan-*rpoB* plasmid for vanillate induction of *rpoB*, we first constructed pCR-*rpoB*-3kb by ligating a 3-kb fragment of *rpoB*^+^ that started at the translation start codon of *rpoB* and that was tailored with a NdeI site into pCR-Blunt. The 3-kb *rpoB*^+^ fragment was then isolated from pCR-*rpoB*-3kb by digestion with NdeI and BamHI and was ligated into the pMRNY3629 vector (Pande et al., 2020) at the NdeI/BglII position, which placed *rpoB* under the control of the vanillate promoter (P_van_).

### Strain Construction

Allele exchange based on the previous protocol (Rodriguez and Spormann, 1999) was carried out to replace the mutant alleles at *Mxan_0795* and *Mxan_6547* in YTY2 with the respective wild-type alleles. YTY2 cells were separately transformed with pBJ-0795 and pBJ-6547, and kanamycin-resistance was used to select for merodiploid transformants in which the plasmid had integrated at the native locus. Eight colonies from each transformation were isolated and grown in CTT liquid in the absence of kanamycin to mid-log phase, and serial dilutions were plated into CTT soft agar containing galactose and incubated at 32°C for 1 week for galactose selection of plasmid excision. All Gal^+^ Kan^s^ colonies bearing the original mutated genetic allele or the intended substituted wild-type allele (strains YTY21 and YTY23) were verified by targeted PCR and sequencing. YTY25 (*0795^+^rpoB^m^6547^+^*) and YTY26 (*0795^+^rpoB^m^6547^m^*) were constructed in the same manner using YTY21 as the parent strain. YTY27 and YTY28 were constructed by transforming YTY25 with pVan-*rpoB* and selecting transformants on plates supplemented with oxytetracycline and 0.5 mM vanillate. YTY27 and YTY28 were generated by homologous recombination downstream and upstream of the *rpoB* mutation, respectively, resulting in chromosomal integration of the plasmid. The desired genetic modifications in YTY27 and YTY28 were verified by targeted PCR and sequencing.

### Vanillate Induction of *rpoB* Expression

To determine the effect of the *rpoB* mutation on RM development, YTY27 and YTY28, expressing the wild-type and the mutated *rpoB*, respectively, along with the control strains (GJV1 and YTY2) were subjected to developmental assays in the presence of 0.5 mM vanillate (Iniesta et al., 2012). Because *rpoB* is an essential gene, vanillate was always added to the medium for YTY27 and YTY28. All the strains (including the controls) were streaked out on CTT-HA plates containing vanillate and incubated at 32°C for 4 days. Strains were then inoculated into CTT liquid supplemented with vanillate. Cultures were grown overnight at 32°C with shaking (300 rpm) until cells entered mid-log phase. The cultures were then centrifuged at 12,000 rpm for 2 min, and pellets were resuspended in TPM liquid to ~5 × 10^9^ cells/ml. Fifty microliters of resuspended cells were then spotted on TPM agar and 0.3%-Casitone CTT agar plates containing vanillate for developmental assays as described below.

### Development and Sporulation Assays

Development and sporulation were performed on starvation (TPM agar) and rich medium (0.3%-casitone CTT-HA) plates. Tested strains were grown from freezer stocks on CTT-HA and incubated at 32°C. The cells were then inoculated into 8 ml CTT liquid and incubated at 32°C, 300 rpm, and collected at mid-log phase for developmental assays. Fifty microliters of the cell suspension (~5 × 10^9^ cells/ml) were spotted onto a TPM-HA plate and a 0.3%-casitone CTT-HA plate, respectively, and then incubated at 32°C for 5 days. Developmental plates were imaged prior to spore collection. Cell spots were harvested into 1 ml ddH_2_O using a sterile scalpel and then incubated at 50°C for 2 h to kill vegetative cells. The samples were then sonicated using a Misonix® Sonicator XL Ultrasonic Processor XL to disperse fruiting bodies and spores. To estimate spore production, serial dilutions of the sonicated samples were plated into CTT soft agar and the plates were incubated at 32°C for 6 days before colonies were counted.

### Growth Measurement at Different Nutrient Levels

Strains tested were grown from freezer stocks on CTT plates and then cultivated in 8 ml CTT liquid at 32°C with shaking (300 rpm) until mid-log phase, at which point cultures were centrifuged, and harvested cells were resuspended to the same cell density (OD_595_~0.15) in CTT liquid containing 0.2%, 0.5%, or 1% casitone. For each cell suspension, 400-μl aliquots were placed in a 48-well plate, with four technical replicates (randomly allocated to minimize the position effect) per strain for each casitone concentration in each biological replicate. The 48-well plate was sealed with air-permeable, transparent plastic film (Greiner Bio-One GmbH, viewseal sealer, clear), incubated in a TECAN plate reader at 32°C and monitored for growth for 300 cycles consisting of the following steps: 5 min shaking (orbital, 2-mm amplitude), 1 min static incubation, and OD_595_ measurement. The values of the three technical replicates were averaged to calculate a biological replicate value, and the same procedure was performed for three biological replicates for each strain.

### Fitness Against a Developmental Cheater

Bacterial cells were collected from a mid-log culture by centrifugation and resuspended to a density of ~5 × 10^9^ cells/ml density in TPM-liquid medium. The kanamycin-resistant cheater strain GJV32 was mixed individually at a 1:99 ratio with GJV1, YTY2, YTY25, or YTY26. Fifty-microliter aliquots of each mix and of each corresponding pure culture were spotted on starvation TPM plates. After 3 days of incubation at 32°C, the plates were harvested as described above for the developmental assays and sonicated samples were dilution plated into CTT soft agar with and without kanamycin. The proportion of germinated cheater cells in the total population count was calculated as the number of kanamycin-resistant spores divided by the total spore count (enumerated on plates without kanamycin).

## Results

### Mutation Identification in the RM Development-Proficient Strain YTY2

**Figure 1:**
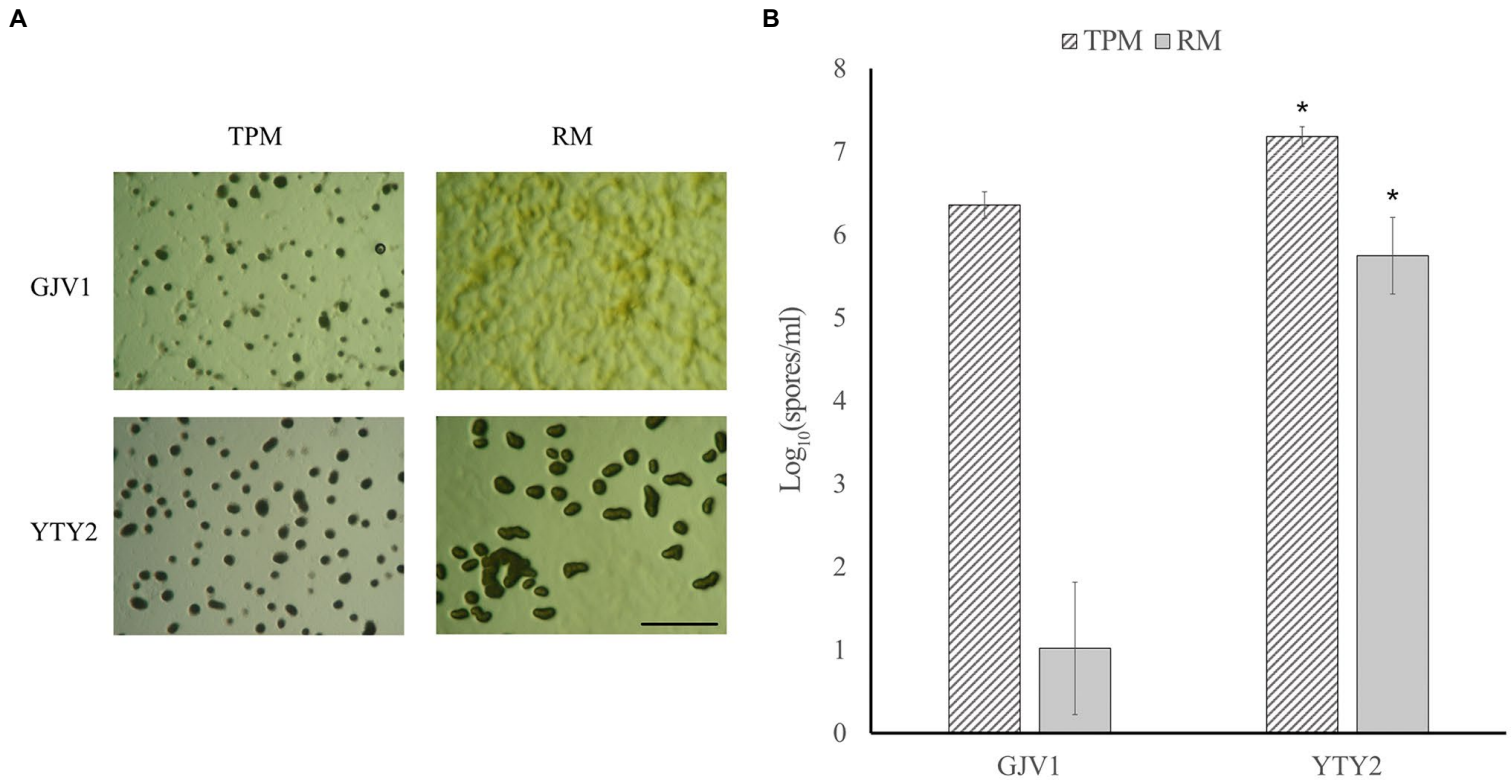
Strain YTY2 is proficient at development on both starvation agar (TPM, 0% casitone) and rich medium agar (RM, 0.3% casitone). **(A)** Developmental morphologies after 5 days on TPM and RM agar. The scale bar is ~1 mm. Dark spots are fruiting bodies. **(B)** Heat-resistant spore production. Error bars show 95% CIs, *n* = 3. Asterisks indicate significant differences compared to GJV1 (*p* = 1.2 × 10^−4^ for TPM and 8.73 × 10^−6^ for RM, paired *t* tests).

In contrast to wild-type GJV1, which cannot initiate development on rich medium (0.3% casitone), YTY2 produces mature fruiting bodies under rich medium conditions in pure culture (Figure 1A). Proficiency at RM development is also evident in sporulation assays (Figure 1B). While GJV1 shows prolific spore production on starvation TPM agar and low to zero spore production on rich medium, YTY2 not only displays significantly higher spore production than GJV1 on TPM but also robust sporulation efficiency on rich medium.

Sanger-sequencing showed that the *pxr* gene of YTY2 was not mutated, indicating that YTY2 RM development is due to mutation of either another known gene in the Pxr pathway or some unknown gene. We therefore sequenced the genome of YTY2 and identified five mutations relative to the published sequence of GJV1 (Velicer et al., 2006). Two mutations found in *Mxan_2515* and *Mxan_7279* could be excluded as candidates for causation of the YTY2 RM development phenotype as they were also found by PCR-based sequencing of the clonal stock of GJV1 from which YTY2 was derived and thus had arisen previously in lab sub-culture. The three remaining mutations resulting in a R253L (CGT → CTT) substitution in *Mxan_0795* (conserved domain protein), S534L (TCG → TTG) in *rpoB* (β-subunit of DNA-directed RNA polymerase, RNAP), and V162I (GTC → ATC) in *Mxan_6547* (TonB-dependent receptor) were considered as candidates for causing RM development.

### The *rpoB* S534L Mutation Is Associated With RM Development

**Figure 2:**
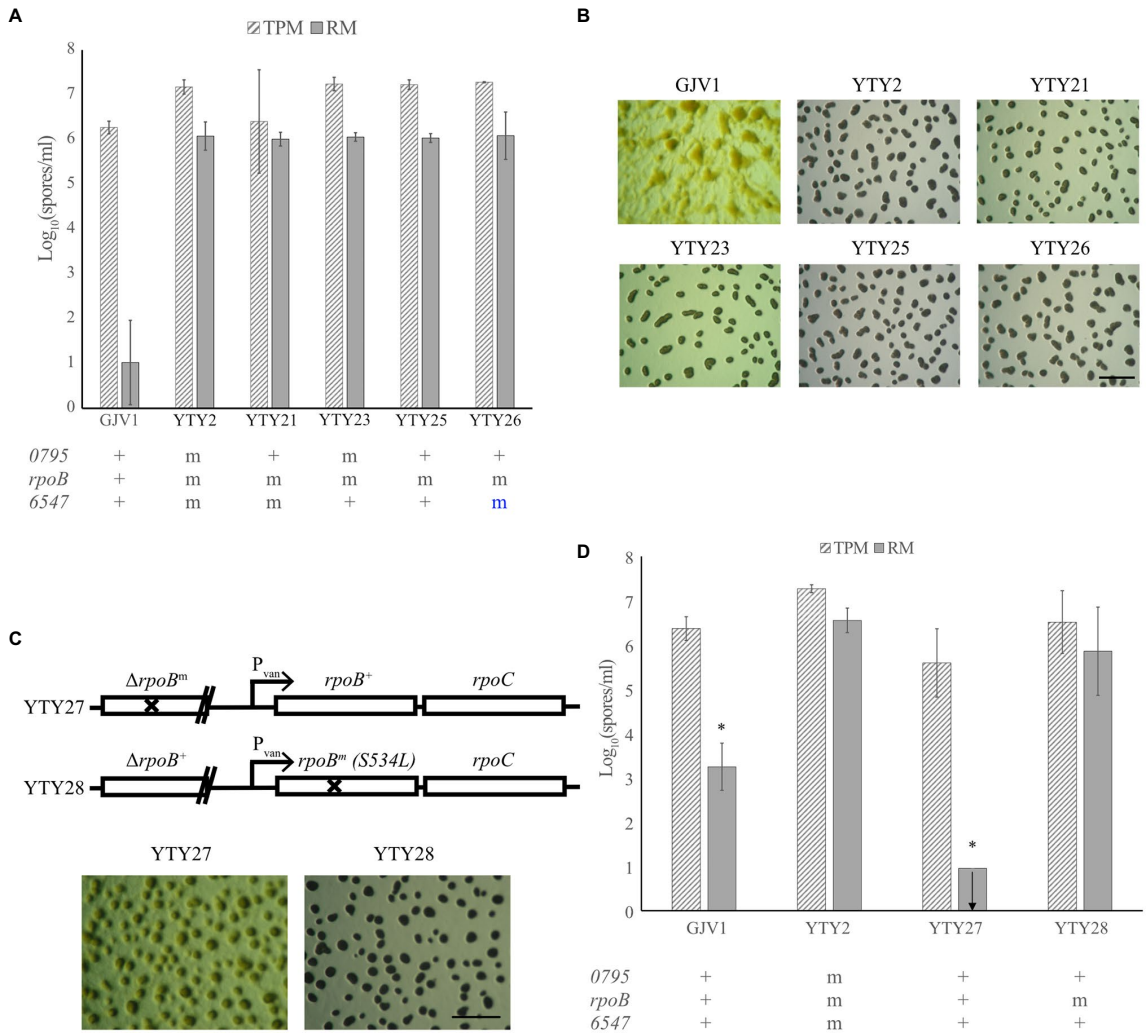
A mutation in *rpoB* is responsible for RM development. **(A)** Spore counts of the different allele-exchanged strains and GJV1 on TPM and RM agar. Each strain’s genetic status at *Mxan_0795*, *rpoB* and *Mxan_6547* is indicated below the graph with “+” designated as the wild-type GJV1 allele and “m” as the mutated version of the gene. Blue “m” letter indicates that the strain retained the mutant allele after the allele exchange process. GJV1 is the only strain significantly different from YTY2 in spore production on RM agar (*p* = 2 × 10^−4^). **(B)** RM developmental phenotypes of the strains shown in panel **(A)**. The scale bar is ~1 mm. **(C)** Genetic layout and RM developmental phenotypes of YTY27 and YTY28. **(D)** Spore counts of strains with the *rpoB* gene under vanillate control compared to YTY2 and GJV1 on TPM and RM agar. YTY27 and YTY28 contain the wild-type and mutant *rpoB* alleles under vanillate control, respectively. GJV1 and YTY27 differ significantly from YTY2 at spore production on RM agar (*p* = 0.0150 and 0.00047, respectively, asterisks, paired *t*-tests; Benjamini–Hochberg (B–H) multiple-testing correction adjusted *α* values 0.0333 and 0.0167, respectively), whereas YTY28 does not. In panels **(A)** and **(D)**, the error bars show the 95% CI; *n* = 3. The arrow indicates that no spores were present at the limit of detection.

To determine which of the three candidate mutations are essential for RM development, allele exchange was used to replace each mutation in YTY2 with the respective wild-type allele. Both YTY21 and YTY23, which have wild-type alleles *Mxan_0795*^+^ and *Mxan_6547*^+^, respectively, remained proficient at RM development (Figures 2A,B), indicating that the mutations in these two loci are not individually required for RM development. Allelic exchange at *rpoB* proved challenging. Because *rpoB* is an essential gene and is transcriptionally coupled to the also-essential downstream gene *rpoC*, the first homologous recombination step of allelic exchange (which creates a merodiploid with two truncated *rpoB* alleles) does not generate viable clones. However, we successfully constructed a strain—YTY25—bearing both *Mxan_0795*^+^ and *Mxan_6547*^+^ while retaining the single-nucleotide TCG → TTG (S534L) mutation in *rpoB* (hereafter also referred to as *rpoB*^m^). Like YTY2, YTY25 produced more spores than GJV1 and exhibited vigorous fruiting body formation in rich medium (Figures 2A,B). As *rpoB*^m^ is the only known genetic difference between GJV1 and YTY25, it seemed likely that RM development is dependent on the S534L substitution in RpoB.

### Expression of the *rpoB*^m^ Allele Confers RM Development

However, it remained possible that genome sequencing of YTY2 failed to detect additional SNPs that contribute to RM development. To eliminate this possibility, we constructed two nearly isogenic strains in the YTY25 background to demonstrate that the *rpoB*^m^ allele is sufficient to confer the RM development phenotype (strain construction is described in Materials and Methods and illustrated in Supplementary Figure S1). Genetic diagrams of the engineered region in those two strains (YTY27 and YTY28) are depicted in Figure 2C. Both strains carry a non-functional, truncated *rpoB* allele under its native promoter and a full-length *rpoB* gene under a vanillate-inducible promoter.

When tested for RM development, some aggregation was observed but no mature fruiting bodies were formed and no sporulation was detected in YTY27 ectopically expressing the *rpoB*^+^ allele under the vanillate-inducible promotor. In contrast, mature fruiting bodies were made and spore production comparable to that of YTY2 was observed in YTY28, which expressed *rpoB*^m^ (Figures 2C,D). From these results, we conclude that RpoB S534L is responsible for enabling RM development.

### *rpoB*^m^ Has Opposite Effects on Growth at Higher vs. Lower Nutrient Concentrations

Although the above experiments showed that *rpoB*^m^ is clearly important for RM development, it remained unclear how the mutant allele altered the normal nutrient-dependent developmental checkpoint on agar medium. One possible scenario is that cells with *rpoB*^m^ erroneously perceive starvation at higher nutrient levels than *rpoB*^+^ cells and thereby trigger the stringent response and subsequent development. In this case, because the stringent response is typically accompanied by growth arrest (Potrykus and Cashel, 2008), liquid cultures of *rpoB*^m^ strains might be expected to arrest growth at nutrient levels at which *rpoB*^m^ strains induce development on agar but wild-type strains do not.

**Figure 3:**
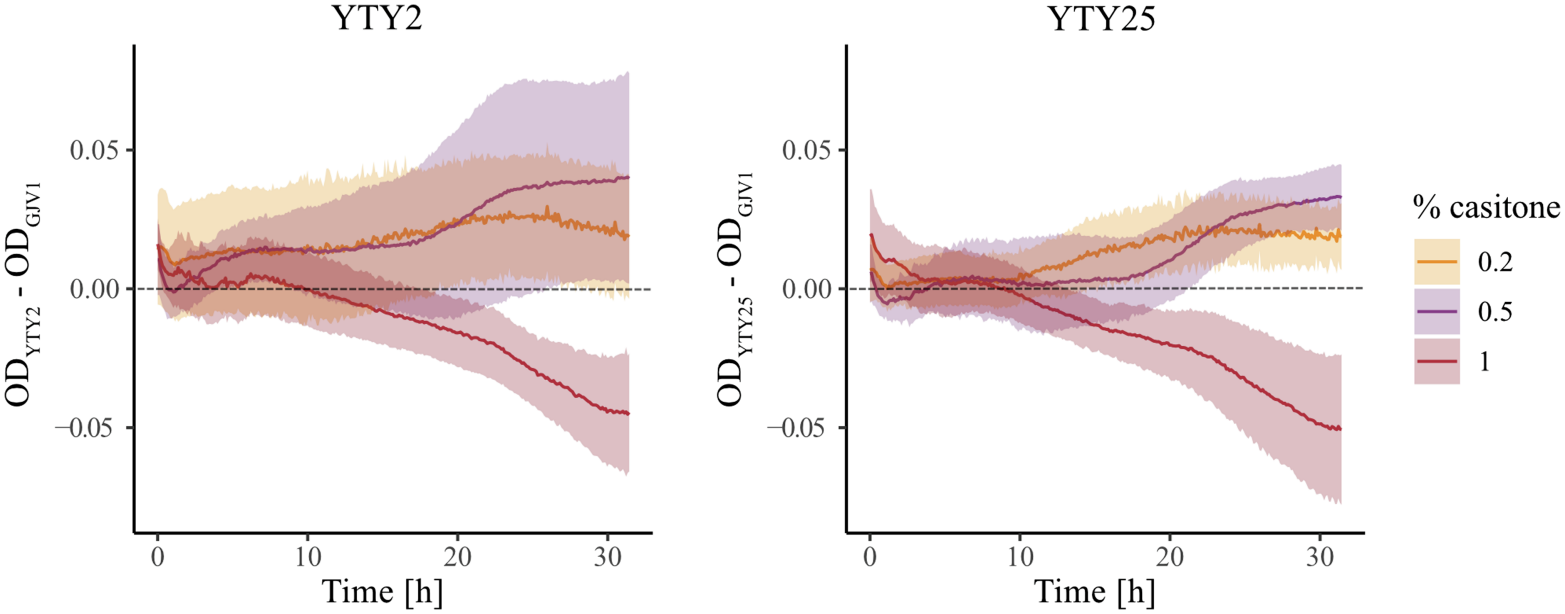
The *rpoB* mutation increases growth rate at lower nutrient levels and decreases growth rate at a higher nutrient level. Differences in OD_595_ between two *rpoB* mutants and GJV1 at three casitone levels over 32 h. Shaded areas indicate 95% confidence regions; *n* = 4.

We monitored growth of GJV1 (*rpoB*^+^), YTY2 (*rpoB*^m^), and YTY25 (*rpoB*^m^) in liquid media at three casitone concentrations (0.2%, 0.5%, and 1.0%), including one concentration (0.2%) below the maximum concentration at which robust development is triggered on agar medium by *rpoB*^m^ genotypes (Figures 1, 2) but not by GJV1. In contrast to the hypothesis above, at 0.2% casitone *rpoB*^m^ cells did not exhibit growth arrest or even growth reduction in liquid medium. In fact the opposite occurred, as YTY2 and YTY25 both grew faster than GJV1 (Figure 3; Supplementary Figure S2). These results from liquid-culture growth suggest that *rpoB*^m^ cells on agar at 0.3% casitone either are able to launch the developmental program without a typical starvation-mediated stringent response or development occurs because the mutant grows faster and thereby depletes nutrients more rapidly under these conditions. The two *rpoB*^m^ strains also grew faster than GJV1 at 0.5% casitone. In contrast, at 1% casitone—the level routinely used to maintain *M. xanthus* vegetative growth—the effect of *rpoB*^m^ on growth reversed direction and the mutants grew slower than GJV1 (Figure 3; Supplementary Figure S2). Thus, the S534L RpoB substitution is found to mediate a tradeoff between growth performance at high vs. low nutrient levels.

### *rpoB*^m^ Suppresses a Developmental Cheater

Strains defective at the sRNA Pxr have been shown both to exhibit RM development (Yu et al., 2010) and to suppress developmental cheating by the obligate cheater strain GJV32 (Fiegna et al., 2006). GJV32 is referred to as a cheater because it is intrinsically defective at development in pure culture but produces disproportionally high numbers of spores in mixed culture with wild-type cells (Velicer et al., 2000). We tested whether *rpoB*^m^ cells can, like a *pxr*-defective strain, similarly prevent cheating by GJV32 despite having a different genetic basis for RM development than *pxr*-defective strains. If so, this might reflect mechanistic similarities in how *rpoB*^m^ and *pxr* mutants cause RM development.

**Figure 4:**
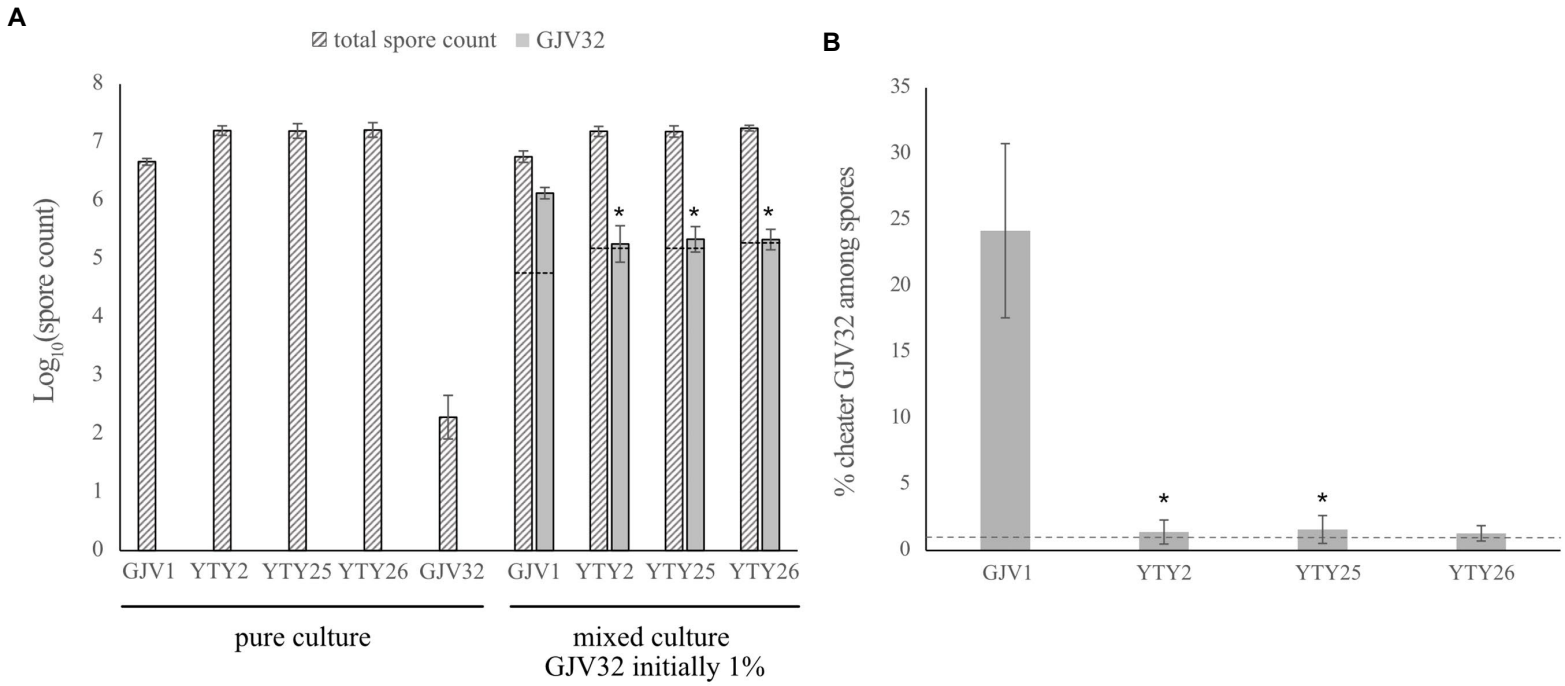
The S534L *rpoB* mutation inhibits developmental cheating. **(A)** Comparison of spore counts between the strains in mono-culture and mixed culture development. The dashed lines indicate expected log values of the GJV32 spore count in mixed cultures initiated with 1% GJV32 if the mono-culture sporulation defect of this strain is fully complemented to the same sporulation efficiency as the developmentally proficient partner, such that 1% of spores in these mixtures derive from GJV32. Error bars show 95% CIs, *n* = 3. Asterisks indicate significant differences of GJV32 spore production relative to mixtures with GJV1 after B–H multiple-testing correction (*p*_YTY2_ = 0.0238, *p*_YTY25_ = 0.0302, and *p*_YTY26_ = 0.0044, paired *t*-tests; B–H adjusted *α* values 0.0333, 0.05, and 0.0167, respectively). **(B)** The cheating efficacy of GV32 on GJV1, YTY2, YTY25, and YTY26. The *y* axis shows the percentage of total spores formed by the cheater GJV32 during development when GJV32 was mixed with four other strains at 1:99 ratio prior to development. The dashed line indicates the percentage of GJV32 spores expected (1%) if GJV32 sporulates at the same efficiency in the mixtures as the other strains. GJV32 makes zero or very few spores in pure culture (Fiegna et al., 2006, GJV32 aka strain “OC”; Fiegna et al., 2006). Error bars show 95% CIs; *n* = 3. Asterisks indicate significant differences of relative GJV32 spore production compared to mixtures with GJV1 after B–H correction (*p*_YTY2_ = 0.0232, *p*_YTY25_ = 0.0186, and *p*_YTY26_ = 0.0176, paired *t*-tests; B–H adjusted *α* values 0.05, 0.0333, and 0.0167, respectively).

The cheater GJV32 was mixed individually with strain GJV1 and with the three strains carrying the *rpoB*^m^ mutation (YTY2, YTY25, and YTY26) at a 1:99 ratio and the mixed populations were subjected to starvation on TPM agar plates. When mixed with GJV1, GJV32 showed the expected cheating phenotype (Figure 4A; Velicer et al., 2000), with GJV32 spores accounting for ~25% of the total viable spores and thus being greatly over-represented compared to the 1% input of the cheater at the beginning of development (Figure 4B). However, this large cheating advantage of GJV32 was absent in mixes with YTY2, YTY25, and YTY26 due to *rpoB*^m^ (Figure 4). The greatly decreased *relative* spore production of GJV32 in mixes with strains carrying *rpoB*^m^ was accomplished because the *rpoB*^m^ strains both produce far more spores than GJV1 and suppress the absolute spore production of GJV32 (relative to its spore production in mixture with GJV1). Because *rpoB*^m^ is the only mutation shared among the three strains and the sole mutation distinguishing YTY25 from GJV1, it is therefore clearly responsible for these effects. Thus, mutations in both the myxobacteria-specific sRNA gene *pxr* and of a broadly conserved subunit of bacterial RNA polymerase can confer RM development and suppress a strong developmental cheater.

## Discussion

*Myxococcus xanthus* prolongs survival during starvation by undergoing multicellular development and generating stress-resistant spores. Tight regulation of this process is important, because only a small percentage of the population differentiates into spores and many cells undergo autolysis (Kroos, 2017). Therefore, undergoing development while nutrients are still abundant would seem undesirable. Previous work from our group has identified several genes that regulate the transition to development, all of which appear to belong to the same regulatory pathway controlled by the small RNA Pxr (Yu et al., 2010, 2016; Chen et al., 2019). In this study, we identify a mutation in the universal bacterial transcription machinery gene *rpoB* that impacts the *M. xanthus* developmental response to nutrients, a connection not revealed by mutations previously known to confer RM development. Specifically, we show that the resulting S534L substitution in RpoB greatly increases the nutrient threshold necessary to prevent development from proceeding (Figure 1). Mutation of both RpoB and RpoD components of the transcription machinery has previously been found to cause fundamental defects in *M. xanthus* development (Rudd and Zusman, 1979; Davis et al., 1995), whereas the S534L substitution in RpoB is found to alter what environmental conditions induce development without causing defects in fruiting body formation and viable spore production. This same mutation is found to also exert complex pleiotropy, impacting multiple other traits. Like *pxr*-defective mutations, *rpoB*^m^ causes suppression of a developmental cheater. Additionally, compared to the parental wild-type, this mutation confers a growth advantage at lower nutrient concentrations and a disadvantage at high nutrient levels.

How does *rpoB*^m^ alter developmental regulation to generate the RM development phenotype? The highly conserved β (RpoB) and β’ (RpoC) subunits of bacterial RNA polymerase contain binding sites for RNA, double-stranded DNA, and DNA/RNA hybrids that play pivotal roles in RNA synthesis (Murakami, 2015). Transcription of *rpoB* does not appear to be strongly regulated early in development, as major changes in *rpoB* expression have not been detected in response to starvation (Muñoz-Dorado et al., 2019). Thus, we consider it unlikely that *rpoB*^m^ mediates RM development by greatly altering cellular RpoB levels.

Starvation stimulates increased concentrations of (p)ppGpp that result in conformational and/or specificity changes in RNAP (Ross et al., 2016). During *M. xanthus* development, the (p)ppGpp-bound RNAP activates early developmental genes, including the genes responsible for A-signal production and genes regulated by A-signal (Harris et al., 1998; Diodati et al., 2014). The *rpoB*^m^ (S534L) mutation in RpoB might cause conformational changes in RNAP similar to those induced by high levels of (p)ppGpp (Zhou and Jin, 1998), such that the altered RNAP is locked into a stringent response-like conformation that stimulates RM development in strains carrying *rpoB*^m^. Consistent with this hypothesis, some *rpoB* mutations in *Streptomyces* species have been found to similarly mimic a stringent response function, namely, antibiotic production that normally depends on starvation-induced (p)ppGpp production (Hu et al., 2002). One such mutation alters the conserved aspartic acid in rifampicin resistance region I of *rpoB* (Hosaka et al., 2009). Mutation of this residue frequently confers resistance to rifampicin in bacteria. The S534L mutation causing RM development in *M. xanthus* lies within the same region just four residues upstream of the conserved aspartic acid, one of the residues that interacts directly with rifampicin (Supplementary Figure S3; Campbell et al., 2001). Interestingly however, the S534L mutation does not confer antibiotic resistance, indicating that at least one change in the rifampicin resistance region I can have major phenotypic effects without altering RNAP structure sufficiently to block rifampicin binding. Moreover, to our knowledge, the specific *rpoB* mutation that changes the conserved serine residue to leucine has never been documented previously in any RpoB homolog.

Alternatively, the *rpoB*^m^ mutation might change the specificity and/or activity of RNAP in a manner that increases growth and thus the rate of nutrient depletion to induce RM development without bypassing dependence on (p)ppGpp. Consistent with this hypothesis, it has been reported that some small deletions in *rpoC* reprogram *E. coli* to grow faster in minimal medium during adaptive evolution (Conrad et al., 2010). In this study, the altered RNAP machinery was found to decrease in open-complex longevity at rRNA promoters, allowing redistribution of RNAP to the rate-limiting amino acid biosynthetic promoters. Additionally, the mutated RNAP reduced transcriptional pausing, resulting in a faster transcriptional elongation rate. Such changes in the kinetic properties of RNAP rewire regulatory gene expression patterns to facilitate optimal growth in a nutrient-limited environment.

That both a mutation in *rpoB* and mutations in the Pxr sRNA pathway can increase the nutrient threshold triggering development raises the intriguing question for future research of whether the mechanistic effects of these distinct mutation categories are related. One hypothesis is that the altered RpoB might act upstream of the Pxr pathway, changing gene expression in a manner that decreases Pxr production. If this were the case, one might expect the *rpoB* mutant examined here and *pxr*-null mutants to be phenotypically similar. However, we have observed several traits distinguishing these mutants. First, when the casitone level is increased to 0.5%, unlike the *pxr*-null mutant (Yu et al., 2010), the *rpoB* mutant no longer makes mature fruiting bodies (Supplementary Figure S4). Second, we have observed that centrifuged cell-culture pellets of the *pxr* mutant are difficult to resuspend in liquid, but pellets of the *rpoB* mutant are not. The greater stickiness of the *pxr* mutant is likely due to increased exopolysaccharide (EPS) production. Third, we have observed that the *rpoB* mutant is tan-colored whereas the *pxr* deletion mutant retains the parental yellow pigmentation. These differences suggest that the *rpoB* mutation may not generate RM development merely by blocking *pxr* expression in a linearly hierarchical regulatory pathway. Alternatively, the *rpoB* mutation and *pxr*-null mutation might independently affect expression of the same gene(s) involved in RM development.

In addition to altering the transition to development, our focal mutation also has strong social effects during development. Specifically, *rpoB*^m^ suppresses a developmental cheater in a manner similar to Pxr-defective strains (Fiegna et al., 2006). In RM development by *rpoB*^m^ genotypes, it is possible that the manner in which *rpoB*^m^ mimics the stringent response eliminates or reduces the need for early extracellular developmental signals normally required to initiate development. Reduced production of such early signals could make *rpoB* mutants less vulnerable to cheating.

Finally, the focal *rpoB*^m^ mutation also strongly impacts vegetative growth. Organisms are often hypothesized to face tradeoffs between competitive abilities in high- vs. low-quality growth environments (Velicer and Lenski, 1999; Pfeiffer et al., 2001; Conrad et al., 2010; Rodríguez-Verdugo et al., 2014). Together with previous studies (Conrad et al., 2010; Rodríguez-Verdugo et al., 2014; Song et al., 2014; Lin et al., 2018; Shiver et al., 2020), our results suggest that *rpoB* has the potential to mediate such tradeoffs. We observed a tradeoff effect of the *rpoB*^m^ mutation on *M. xanthus* growth between higher vs. lower nutrient media, as the mutation increases growth relative to the parental genotype in lower nutrient media while slowing growth in richer media (Figure 3). A similar pattern was observed by Lin et al. (2018) who found that the competitiveness of rifampicin-resistant *rpoB* mutants of *E. coli* and *Pseudomonas aeruginosa* relative to their wild-type parents correlated inversely with the nutrient concentration of competition media. In a different form of stress-related tradeoff, an *rpoB* mutation in *E. coli* was found to increase growth at stressful high temperatures while decreasing fitness at lower temperatures (Rodríguez-Verdugo et al., 2014).

That a mutation in *rpoB* rewires the first steps of myxobacterial development raises intriguing questions about what roles this ancient gene has played in the emergence of clade-specific forms of environmental stress response. That the same mutation pleiotropically impacts several aspects of the *Myxococcus* life cycle, including vegetative growth and social interactions during development, raises additional questions regarding potential evolutionary interactions between the general transcriptional machinery and the evolution of complex life histories. Such complex pleiotropy suggests that *rpoB* may often be a joint target of diverse selective forces operating through the myxobacterial life cycle, including social forces unique to the myxobacteria and abiotic forces experienced by all prokaryotes.

## Data Availability Statement

The datasets presented in this study can be found in online repositories. The names of the repository/repositories and accession number(s) can be found at: https://www.ncbi.nlm.nih.gov/, PRJNA779264. Dryad Digital Repository available at: https://doi.org/10.5061/dryad.jsxksn0b0.

## Author Contributions

SE and Y-TY executed experiments. Y-TY designed research. SE, Y-TY, and GV analyzed the data and wrote the paper. All authors contributed to the article and approved the submitted version.

## Funding

This work was funded in part by Swiss National Science Foundation (SNF) grant 310030B.

## Conflict of Interest

The authors declare that the research was conducted in the absence of any commercial or financial relationships that could be construed as a potential conflict of interest.

## Publisher’s Note

All claims expressed in this article are solely those of the authors and do not necessarily represent those of their affiliated organizations, or those of the publisher, the editors and the reviewers. Any product that may be evaluated in this article, or claim that may be made by its manufacturer, is not guaranteed or endorsed by the publisher.
