# Supplementary material for: Mutation of rpoB Shifts the Nutrient Threshold Triggering Myxococcus Multicellular Development
Source: Front Microbiol. 2022 Mar 3;13:817080. doi: 10.3389/fmicb.2022.817080 (PMC8963815; doi:10.3389/fmicb.2022.817080)
Supplement: Supplementary file 1 [file Data_Sheet_1.docx]

Supplementary Material

# Supplementary Figures and Tables

## Supplementary Tables

**Table S1. Strains.**

| **Strain** | **Description** | **Reference** |
| --- | --- | --- |
| Top10 | *E. coli* strain for plasmid construction | Invitrogen |
| GJV1 | Wild-type *M. xanthus* lab strain, a derivative of DK1622 | (Velicer et al., 2006) |
| GJV2 | Rifampicin-resistant variant of GJV1 | (Fiegna et al., 2006) |
| GJV32 | Developmental cheater | (Velicer et al., 2006) |
| YTY2 | A derivative of GJV1 containing mutations in *Mxan_0795*, *rpoB* (*Mxan_3077*), and *Mxan_6547* is able to develop in rich medium | This study |
| YTY21 | YTY2 substituted with *Mxan_0795*^+^ allele.  *YTY2::0795^+^* | This study |
| YTY23 | YTY2 substituted with *Mxan_6547*^+^ allele.  YTY2::*6547^+^* | This study |
| YTY25 | *YTY21 substituted with Mxan_6547^+^ allele. YTY2::0795^+^ 6547^+^* | This study |
| YTY26 | YTY21 underwent allele exchange but kept the mutation in *Mxan_6547.* YTY2::*0795^+^ 6547^m^* | This study |
| YTY27 | YTY25 transformed with pVan-*rpoB* results in expression of *rpoB^+^* allele under vanillate induction. | This study |
| YTY28 | YTY25 transformed with pVan-*rpoB* results in expression of *rpoB^m^* allele under vanillate induction. | This study |

**Table S2. Primers.**

| **Primer** | **Sequence** | **Description** |
| --- | --- | --- |
| GV690 | AGTTCAACAGAGCCCGCCGCG | 0795f |
| GV691 | AGCCATGCGCGGCCACGGTAT | 0795r |
| GV692 | GTGCATGGACGCGCCCGTGG | 7279f |
| GV693 | AGCAGCCCCGCCAGGCCCTG | 7279r |
| GV704 | GACAACCTCAACGTGGGCCCGTACC | *rpoB*f |
| GV705 | AGGAACGGGATGAGCGACGCGGC | *rpoB*r |
| GV697 | GCGATGAACGCGGAGGAGATT | 6547f |
| GV698 | GTCCAGCCGCGAGTTGCCACC | 6547r |
| GV702 | ATGGTGGGCAACTCCTTGCAGGG | 2515f |
| GV703 | GAGATCTTCCCGCGCACGTTCTCC | 2515r |
| GV802 | AACATATGCCGACGCAGATCCAGAACAA | 3-kb *rpoB*f |
| GV803 | TTCTTGATGCGGGCGATCTTCTC | 3-kb *rpoB*r |

**Table S3. Plasmids.**

| **Plasmid** | **Description** | **Reference** |
| --- | --- | --- |
| pCR-Blunt | Cloning/integrative vector | Invitrogen |
| pMRNY3629 | A derivative of pMR3629 containing a vanillate-inducible expression cassette and an oxytetracycline resistance marker | (Pande et al., 2020) |
| pCR-0795 | ~500-bp PCR fragment of *Mxan_0795* amplified with primers GV690 and GV691 cloned into pCR-Blunt | This study |
| pCR-6547 | ~1000 bp PCR fragment of *Mxan_6547* amplified with primers GV697 and GV698 cloned into pCR-Blunt | This study |
| pCR-*rpoB* | ~1000 bp PCR fragment of *rpoB* amplified with primers GV704 and GV705 cloned into pCR-Blunt | This study |
| pCR-*rpoB*-3kb | ~3000 bp 5’ terminal fragment of *rpoB* amplified with primers GV802 and GV803 cloned into pCR-Blunt | This study |
| pBJ113-*cglB* | Vector for allele exchange carrying a partial *cglB* gene, a kanamycin resistance cassette and the galactokinase gene(*galK*) | (Rodriguez and Spormann, 1999) |
| pBJ-0795 | ~500 bp PCR fragment of *Mxan_0795* replaces the *cglB* portion of pBJ113-*cglB* | This study |
| pBJ-6547 | ~1000 bp PCR fragment of *Mxan_6547* replaces the *cglB* portion of pBJ113-*cglB* | This study |
| pBJ-*rpoB* | ~1000 bp PCR fragment of *rpoB* replaces the *cglB* portion of pBJ113-*cglB* | This study |
| pVan-*rpoB* | a 3 kb fragment of *rpoB* (GJV1 allele) cloned into pMRNY3629 and placed under a vanillate-inducible promoter | This study |

## Supplementary Figures


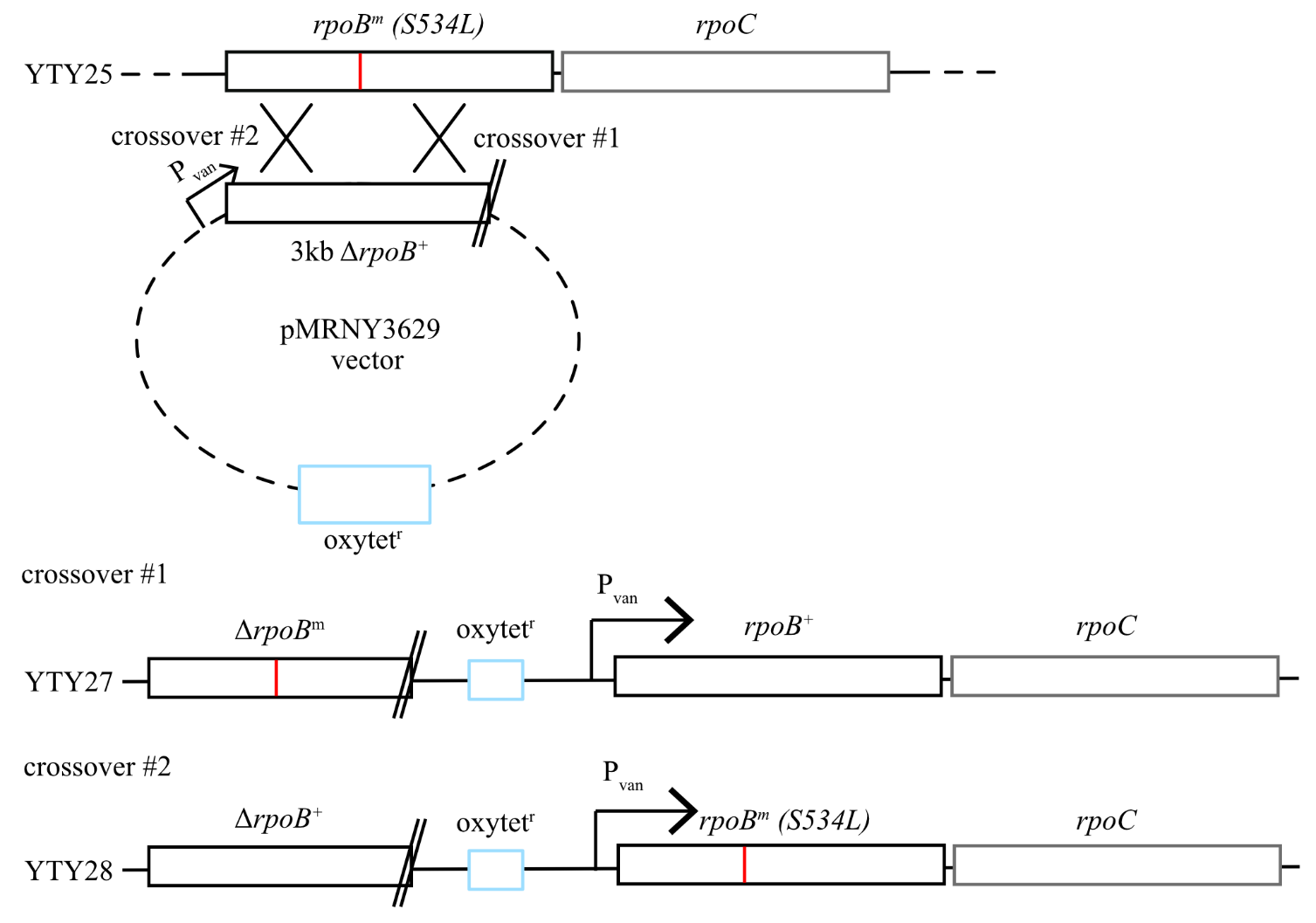
 **Figure S1.** Construction of YTY27 and YTY28. Insertion of pVan-*rpoB* by homologous recombination at the *rpoB* locus of strain YTY25 leads to two reciprocal strains, where *rpoB^+^* (YTY27) or *rpoB^m^* (YTY28) is expressed under a vanillate-inducible promoter, depending on whether the crossover occurred before (YTY28) or after (YTY27) the position of the *rpoB* mutation.


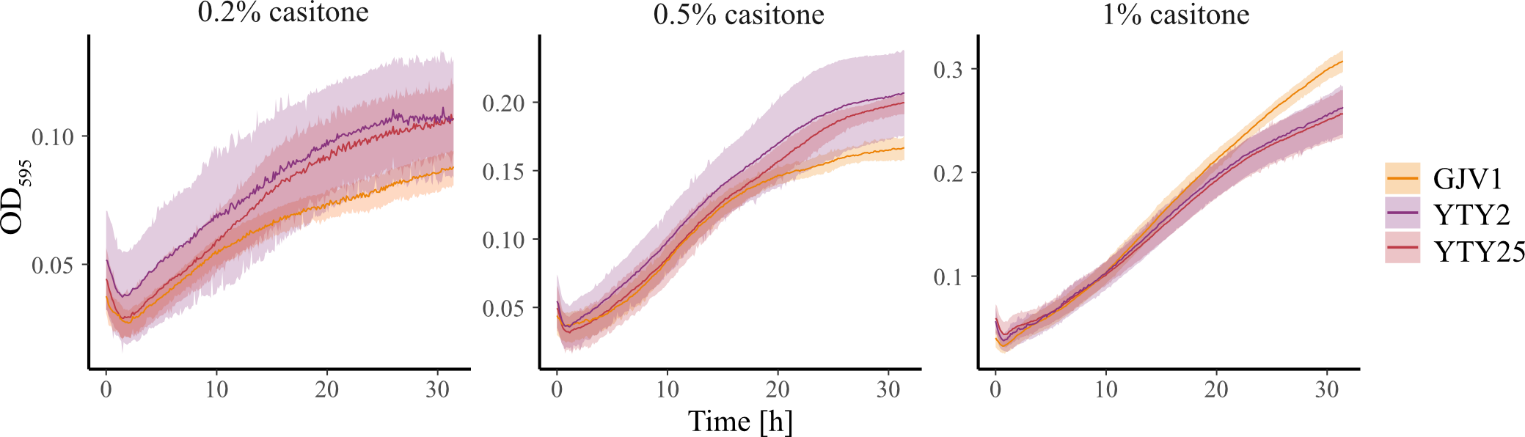


**Figure S2.** The *rpoB* mutation increases growth at lower nutrient levels and decreases growth at a higher nutrient level. Original OD_595_ values for two *rpoB* mutants and GJV1 at three casitone levels over 32 h. Shaded areas indicate 95% confidence regions; n = 4.


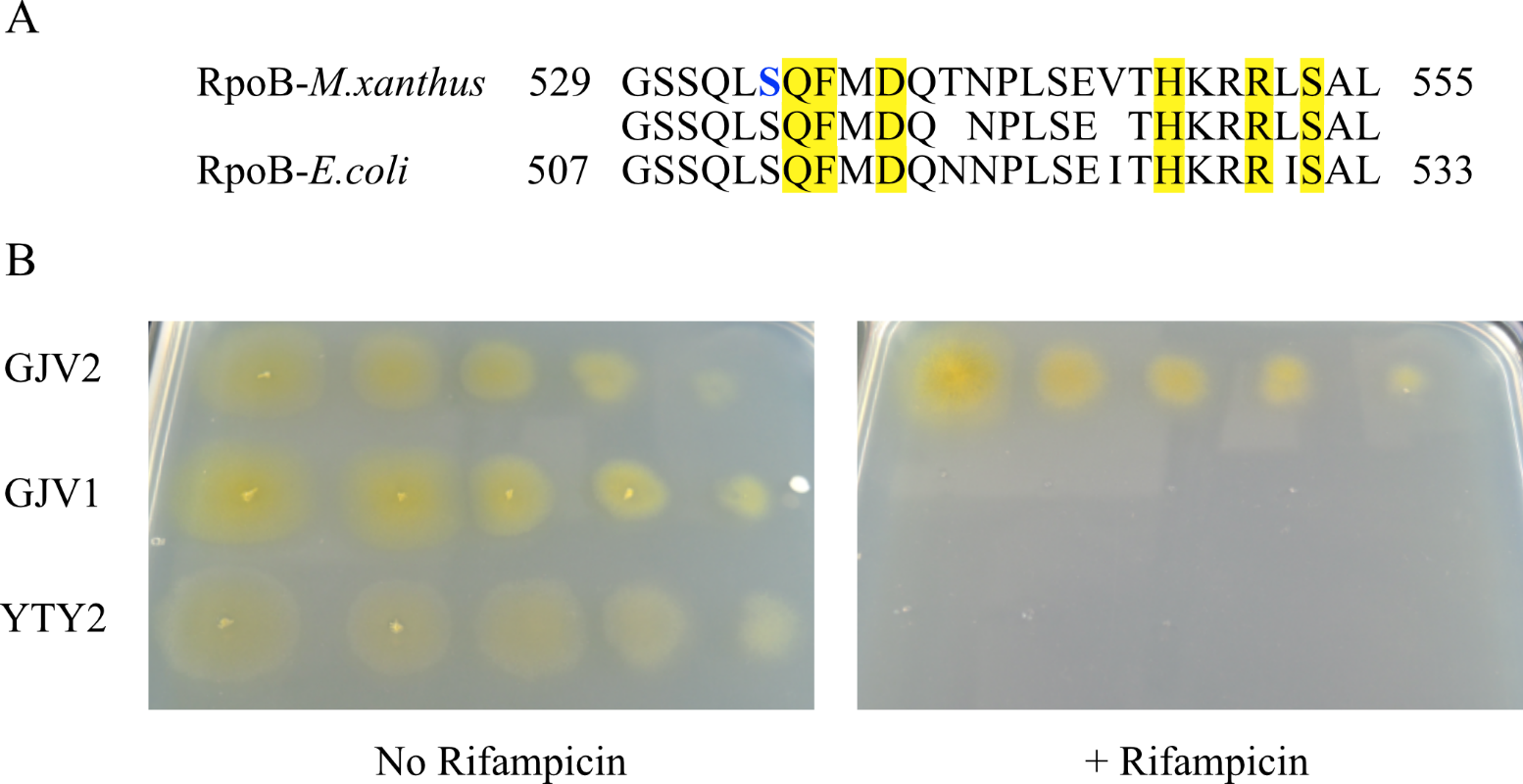


**Figure S3**. **(A)** The S534L RpoB substitution in strain YTY2 is located in the designated rifampicin-resistance region I. Residues participating directly in rifampicin binding are highlighted in yellow in the shown alignment of the *M. xanthus* and *E. coli* RpoB rif-resistance regions (Campbell et al., 2001). The residue S534 examined in this study is shown in blue. **(B)** The S534L RpoB substitution in YTY2 does not confer rifampicin resistance. Mid-log cultures of GJV1 (the wild-type parental strain), GJV2 (the rif-resistant reference strain) and YTY2 (S534L change in RpoB) were serially diluted by a factor of 10 and 2 μl of each diluted sample was spotted on CTT hard agar containing either 0 or 5 μg/ml rifampicin (left and right images, respectively). The original uncropped images were deposited in Dryad.


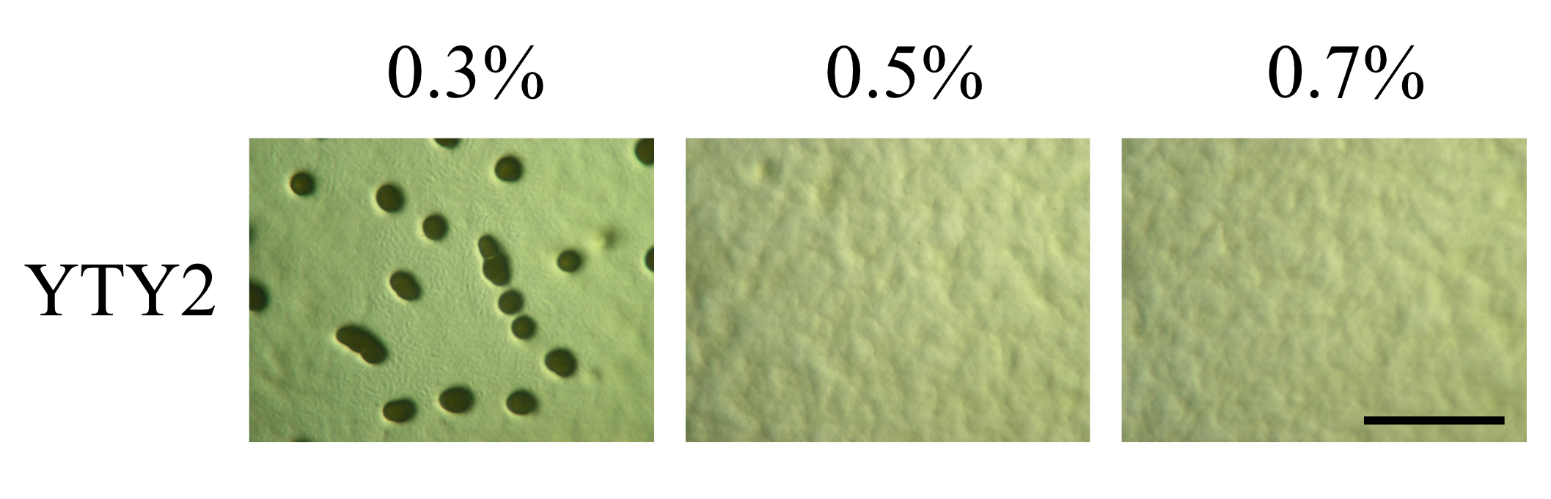


**Figure S4.** YTY2 does not develop at higher nutrient levels. YTY2 was examined for fruiting body formation on nutrient-agar plate containing 0.3%, 0.5% and 0.7% casitone. The scale bar is ~1 mm.
